# Case Report: Nicotinamide monotherapy for infantile bullous pemphigoid with long-term follow-up

**DOI:** 10.3389/fimmu.2026.1935375

**Published:** 2026-09-21

**Authors:** Peng Qu, Guizhi Zhou, Baoqi Yang

**Affiliations:** 1 Dermatology Hospital of Shandong First Medical University, Jinan, Shandong, China; 2 Shandong Provincial Institute of Dermatology and Venereology, Shandong Academy of Medical Sciences, Jinan, Shandong, China

**Keywords:** autoimmune blistering disease, infantile bullous pemphigoid, long-term follow-up, monotherapy, nicotinamide

## Abstract

Infantile bullous pemphigoid (IBP) is a rare autoimmune bullous skin disease that occurs in infants, for which standardized targeted therapeutic regimens remain unavailable. Herein, we report two infants with IBP treated with nicotinamide monotherapy. Complete clinical remission was observed within 3 weeks and 10 days after treatment initiation, respectively. Following clinical improvement, nicotinamide was gradually tapered and discontinued in approximately 2 months. No recurrence was observed during long-term follow-up. However, given the limited number of cases, these observations do not establish the therapeutic efficacy of nicotinamide but provide clinical experience with nicotinamide monotherapy in IBP.

## Introduction

Infantile bullous pemphigoid (IBP) is a rare autoimmune subepidermal bullous disease characterized by the production of autoantibodies against the BP180 antigen (1). Distinct from adult-onset bullous pemphigoid (BP), IBP typically manifests as acral blistering with rare mucosal involvement and generally carries a favorable prognosis (2). Systemic corticosteroids remain the first-line therapy for IBP; however, their clinical application in infants is limited by well-documented adverse effects, highlighting the demand for safer alternatives. In this study, we report two cases of IBP treated with nicotinamide monotherapy, both of which achieved rapid and sustained clinical remission without recurrence during long-term follow-up.

## Case 1

A 2-month-old male infant presented to our clinic with a 17-day of generalized erythematous macules and 4-day of vesicles. He was delivered via spontaneous vaginal birth. He received Bacillus Calmette–Guérin (BCG) vaccination and the first dose of hepatitis B vaccine within 24 hours postpartum, followed by the second dose of hepatitis B vaccine at 1 month of age. Twenty days before the lesions appeared, he completed the second hepatitis B vaccination. He was initially diagnosed with erythema multiforme at a local pediatric outpatient clinic and received a 5-day course of hydrocortisone at a daily dose of 3.5 mg, with no obvious clinical improvement. Physical examination revealed generalized erythematous patches, tense vesicles and crusted lesions distributed over the trunk and extremities (**Figure 1A**), with a negative Nikolsky sign. No familial history of hereditary skin or autoimmune diseases was reported by the parents.

**Figure 1:**
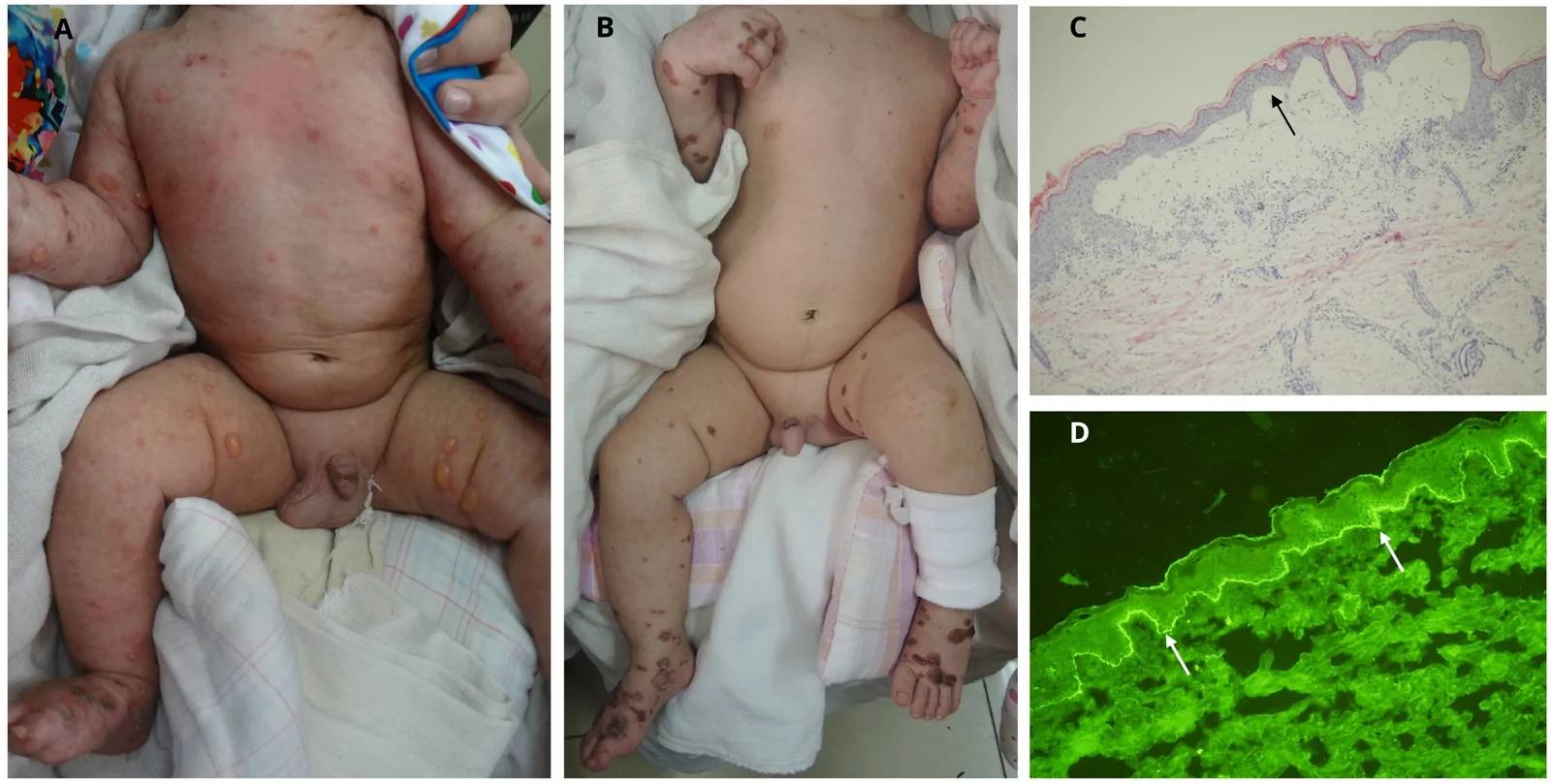
Clinical, histopathological, and immunofluorescence findings in case 1. **(A)** Multiple erythematous patches and tense vesicles distributed over the face, trunk, and extremities; vesicles measured up to 1 cm in diameter. The Nikolsky sign was negative. **(B)** After treatment, all blisters desiccated and crusted over, with no new cutaneous lesions emerging. **(C)** Histopathological examination showing a subepidermal blister (black arrows) with scattered eosinophils in the superficial dermis (H&E staining; original magnification, ×100). **(D)** Direct immunofluorescence demonstrating linear C3 deposition along the basement membrane zone (white arrows) (original magnification, ×200).

Laboratory examinations showed an elevated C-reactive protein level of 29.78 mg/L and a white blood cell count of 13.67 × 10^9^/L, with neutrophils accounting for 43.6% of the total leukocytes. Eosinophil count, serum IgE level and other inflammatory biomarkers were all within normal reference ranges. Histopathological analysis of lesional skin biopsy specimens revealed subepidermal blister formation accompanied by scattered eosinophils (**Figure 1C**). Direct immunofluorescence (DIF) staining showed linear C3 deposition along the epidermal basement membrane zone (BMZ) (**Figure 1D**). Neither conventional indirect immunofluorescence (IIF) for circulating pemphigoid autoantibodies nor salt-split skin IIF for circulating IgG antibodies yielded positive results at an initial serum dilution of 1:10. However, the serum anti-BP180 antibody titer was markedly elevated at 90 U/mL (positive cutoff value: 9 U/mL).

Based on the clinical manifestations, histopathological features, immunofluorescence findings and serological results, a definitive diagnosis of IBP was established. Oral nicotinamide monotherapy was initiated at a dosage of 50 mg three times daily (total daily dose: 150 mg, 30 mg/kg/day). The formation of new blisters ceased rapidly after treatment initiation, and complete clinical remission was achieved within 3 weeks (**Figure 1B**). Nicotinamide treatment was then discontinued in two months, and the patient remained disease-free without recurrence during a 10-year follow-up period.

## Case 2

An 8-month-old male infant presented to our department with a 2-week history of pruritic vesicles and bullae predominantly affecting the feet, with mild involvement of the hands (**Figures 2A, B**). His parents reported that he had received a diphtheria and tetanus (DT) vaccine 9 days before the onset of the skin lesions. No preceding systemic infection or family history of hereditary diseases was reported. Prior to our consultation, the patient had received oral loratadine and topical corticosteroid ointment at a local outpatient clinic, with no significant symptomatic relief or lesion regression.

**Figure 2:**
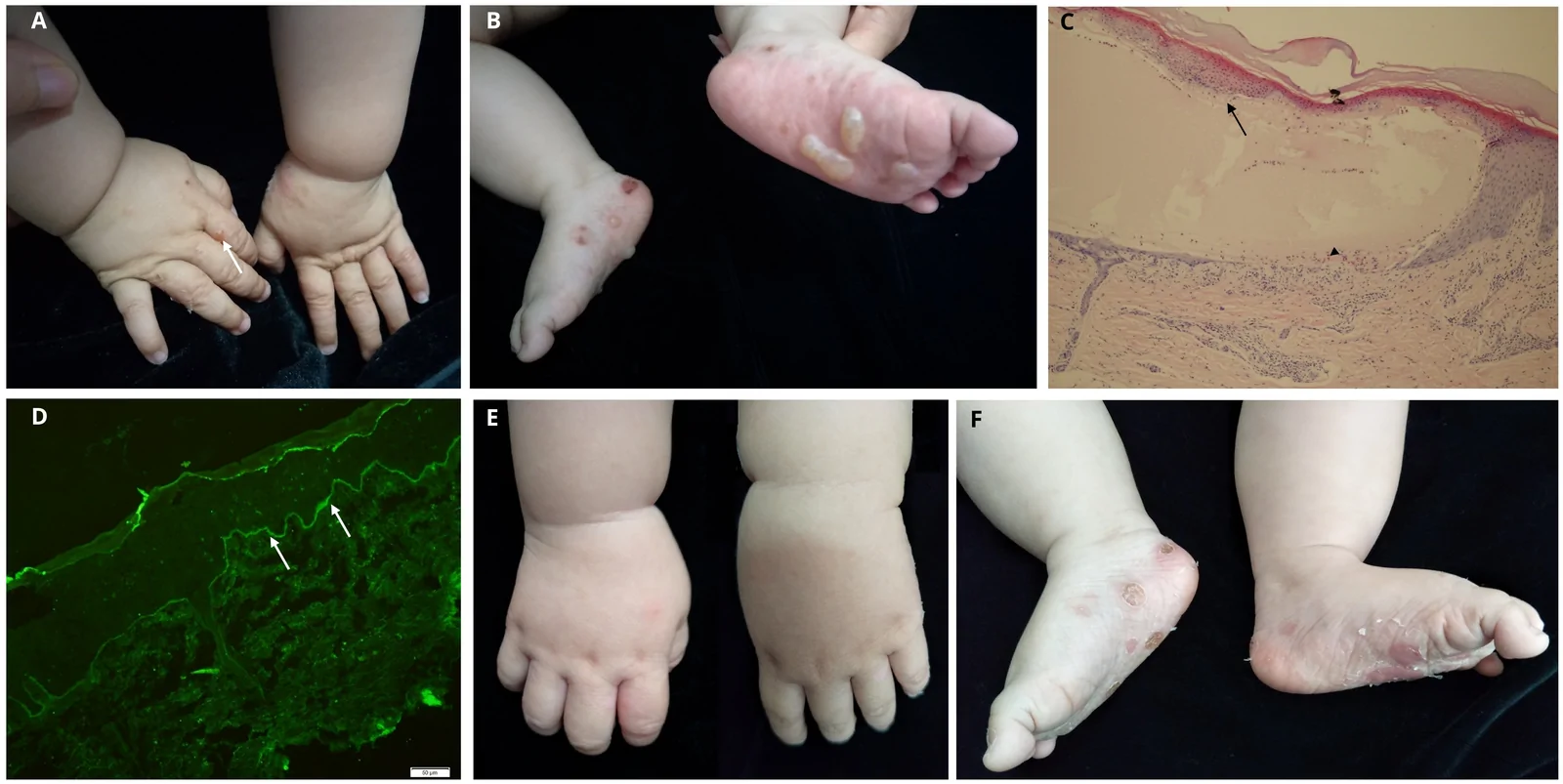
Clinical, histopathological, and immunological findings in case 2. **(A, B)** Mild involvement of the hand, with a small vesicle (white arrow). More prominent vesiculobullous lesions involving the foot without mucosal involvement; the Nikolsky sign was negative. **(C)** Histopathological examination showing a subepidermal blister (black arrow) with scattered eosinophils in the superficial dermis (black arrowheads) (H&E staining; original magnification, ×100). **(D)** Direct immunofluorescence demonstrating linear deposition of IgG (white arrows) along the basement membrane zone (original magnification, ×200). **(E, F)** After treatment, erythema on palms and soles resolved, all blisters desiccated and crusted without new blister formation.

Physical examination revealed multiple pruritic vesicles and tense bullae predominantly involving the feet, with only mild involvement of the hands, where a small vesicle was observed. Nikolsky’s sign was negative, and no vesicles or erosions were observed on the oral mucosa. Laboratory tests revealed peripheral eosinophilia (12.3%; absolute eosinophil count: 0.94 × 10^9^/L) and an elevated serum anti-BP180 antibody level of 77 U/mL. Histopathological examination confirmed subepidermal blister formation with prominent eosinophilic infiltration (**Figure 2C**). DIF staining revealed linear deposition of both IgG and C3 along the BMZ (**Figure 2D**), and IIF testing detected circulating pemphigoid autoantibodies with linear BMZ fluorescence at a serum dilution of 1:10.

Based on the comprehensive clinical, histopathological, immunopathological and serological evidence, the patient was diagnosed with IBP. Oral nicotinamide monotherapy was administered at a dosage of 50 mg three times daily (total daily dose: 150 mg, 18.75 mg/kg/day), and complete clinical remission was achieved within 10 days of treatment (**Figures 2E, F**). Nicotinamide was subsequently discontinued in two months, and the patient maintained sustained complete remission without any disease recurrence during a 9-year follow-up period.

## Discussion

IBP is a rare autoimmune subepidermal blistering disorder characterized by autoantibodies targeting hemidesmosome proteins, predominantly BP180 and BP230. IBP presents distinct clinical characteristics compared with adult-onset BP. Typical manifestations include localized acral lesions with rare mucosal involvement, and most patients achieve complete remission with a favorable overall prognosis (1, 2). These clinical features were consistent with our two enrolled patients, particularly the second case, whose skin lesions were strictly limited to the hands and feet.

The potential association between vaccination and IBP onset remains controversial. In a review of 81 reported cases of infantile BP, 25 patients (25/81, 30.8%) had a history of vaccination before disease onset, with the reported interval between vaccination and disease onset ranging from 1 day to 4 weeks (1). Both of our patients had a history of recent vaccination before disease onset: Case 1 developed skin lesions 20 days after hepatitis B vaccination, whereas Case 2 developed lesions 9 days after receiving a DT vaccine. These temporal associations are compatible with previously reported latency periods; however, they do not establish a causal relationship between vaccination and IBP. Vaccination is hypothesized to act as an immunological trigger for IBP in genetically susceptible infants. The underlying mechanisms may involve the activation of the immature infant immune system by vaccine antigens and adjuvants, upregulated production of pro-inflammatory cytokines, and T cell-mediated activation of autoreactive B cells, which ultimately induce the generation of pathogenic anti-BP180 autoantibodies (3–5). Nevertheless, current epidemiological evidence is insufficient to confirm a definitive causal relationship between vaccination and IBP.

The diagnosis of IBP is challenging due to its overlapping clinical manifestations with multiple infantile blistering dermatoses, including linear IgA bullous dermatosis (LABD), epidermolysis bullosa acquisita (EBA), hereditary epidermolysis bullosa, staphylococcal scalded skin syndrome, and other autoimmune or inflammatory blistering diseases. Our first patient was initially misdiagnosed with erythema multiforme at a local pediatric clinic. However, subsequent histopathological examination identified subepidermal blisters with eosinophilic infiltration, DIF detected linear C3 deposition at the BMZ, and serological testing revealed significantly elevated anti-BP180 antibody titers, all of which supported the diagnosis of IBP. Of note, conventional IIF and salt-split IIF were negative despite a positive anti-BP180 ELISA (90 U/mL). This discordance may reflect methodological differences between the assays, as BP180 ELISA specifically detects antibodies against the NC16A domain, whereas the sensitivity of IIF can be influenced by the substrate, antigen accessibility, and circulating antibody levels. Nevertheless, the diagnosis was supported by the characteristic clinical and histopathological findings, linear C3 deposition along the BMZ on DIF, and positive anti-BP180 serology. The lesions of the second patient were confined to the extremities, clinically mimicking LABD and other localized blistering disorders. Nonetheless, histological examination showed subepidermal blisters with marked eosinophilic infiltration, while DIF demonstrated linear IgG and C3 deposition at the BMZ, combined with positive IIF results and elevated anti-BP180 antibodies. These findings collectively supported the diagnosis of IBP. Collectively, these cases indicate that the accurate diagnosis of infantile vesiculobullous diseases requires comprehensive integration of clinical manifestations, histopathological features, immunofluorescence results and antigen-specific serological tests, rather than reliance on a single diagnostic index (6, 7).

Systemic corticosteroids, either alone or in combination with dapsone, are among the most commonly reported treatments for infantile BP, although no universally standardized treatment regimen has been established. We elected to treat the two infants with nicotinamide primarily because of their very young age and our aim to minimize exposure to systemic corticosteroids and their potential adverse effects. Our treatment decision was also informed by our previous clinical experience with nicotinamide in childhood LABD. In a previous report from our group, a 22-month-old child with LABD achieved complete clinical remission within 7 days of oral nicotinamide monotherapy, without recurrence during 10 months of follow-up (8). Although LABD and IBP are distinct autoimmune blistering disorders with different target antigens and immunopathogenic mechanisms, this prior clinical experience encouraged us to consider nicotinamide monotherapy as an option in these two infants. In addition, nicotinamide has previously been investigated as alternative treatment for BP, and its anti-inflammatory effects have been well described. As early as 2002, the British Association of Dermatologists (BAD) guidelines recommended tetracycline combined with nicotinamide as a first-line alternative regimen for localized and mild-to-moderate BP, based on evidence from small randomized controlled trials and clinical case series (9, 10). This combination regimen was retained in the updated 2012 BAD guidelines (11). However, most existing evidence supports the efficacy of combined therapy in adult patients, with limited data on nicotinamide monotherapy. Notably, tetracycline antibiotics are contraindicated in infants due to the risk of irreversible dental enamel damage. To date, the clinical efficacy and safety of nicotinamide monotherapy for IBP remain poorly defined.

The potential effects of nicotinamide in BP may be related to its anti-inflammatory and immunomodulatory properties. Nicotinamide has been reported to inhibit leukocyte chemotaxis and secretion, including those of neutrophils and eosinophils, and to exert additional anti-inflammatory effects through inhibition of mast-cell degranulation and histamine-related responses (12, 13). These effects provide a plausible mechanistic rationale for its potential use in BP, suggesting that nicotinamide may primarily attenuate the downstream inflammatory effector phase following BP180 autoantibody binding rather than directly suppress BP180-specific autoantibody production. However, these mechanisms are largely inferred from experimental and pharmacological studies, and the precise immunomodulatory effects of nicotinamide in BP, particularly in infantile BP, remain to be elucidated.

Our two cases provide preliminary clinical experience with nicotinamide monotherapy in IBP. An important consideration when interpreting these observations is the favorable natural history of the disease. In a systematic review of 81 reported cases, all but one patient eventually achieved remission, indicating an overall favorable prognosis (1). However, most patients received active treatment, predominantly systemic corticosteroids alone or in combination with dapsone or sulfapyridine; therefore, these data cannot be used to estimate the spontaneous remission rate or its typical time course in untreated IBP. Spontaneous remission has nevertheless been documented. Nomura et al. reported an infant with generalized BP whose parents declined corticosteroid treatment and who was managed with supportive topical care alone; the disease resolved spontaneously within approximately 4 months, with no recurrence during 7 years of follow-up (14). In comparison, complete clinical remission was observed within 3 weeks and 10 days after initiation of nicotinamide monotherapy in our two patients, respectively. Although this relatively rapid temporal response is clinically noteworthy and suggests a possible treatment-associated effect, spontaneous improvement cannot be excluded.

Of note, the two cases presented distinct lesion distributions: the first patient exhibited generalized cutaneous involvement, while the second had strictly localized acral lesions. Currently, there are no standardized treatment protocols or optimal dosage guidelines for nicotinamide in IBP management. In our patients, the initial dose of 150 mg/day (50 mg three times daily) was selected empirically based on our clinical experience, the patients’ body weights, and the available tablet strength of nicotinamide (50 mg per tablet), corresponding to 30 mg/kg/day in Case 1 and 18.75 mg/kg/day in Case 2. Thus, the same absolute daily dose represented an individualized clinical decision rather than a standardized weight-based dosing regimen. It is noteworthy that Case 2, who received the lower weight-adjusted dose, also had relatively mild and localized disease with fewer blisters; however, this was a retrospective observation, and no relationship between disease severity and the required nicotinamide dose can be inferred from these two cases. Following clinical improvement, nicotinamide was gradually tapered according to the individual clinical course of each patient and discontinued in 2 months. Nevertheless, the observational nature of these two cases limits the ability to validate the definitive efficacy and optimal dose and treatment duration of nicotinamide for IBP.

In recent years, the therapeutic options for IBP have expanded beyond conventional systemic corticosteroids. Dapsone has been validated as an effective steroid-sparing agent for pediatric BP (2). Intravenous immunoglobulin (IVIG) serves as a rescue therapy for severe or refractory IBP cases (15, 16). Cyclosporine is another alternative for refractory IBP, with a successful refractory case reported by Bar-Ilan et al. (17). Additionally, baricitinib, a Janus kinase (JAK) inhibitor, has emerged as a novel therapeutic option for highly refractory IBP in recent years (18). However, clinical evidence supporting the use of cyclosporine and baricitinib in IBP remains scarce and limited to individual case reports, and their application requires individualized clinical assessment.

This study has several limitations. First, the small sample size (only two cases) precludes comparative analysis with systemic corticosteroids or other steroid-sparing regimens, and we cannot establish the superiority of nicotinamide monotherapy. Second, the optimal dosage and treatment duration of nicotinamide for IBP remain undetermined. In addition, serum anti-BP180 antibody levels were not reassessed after treatment in either patient; therefore, we were unable to evaluate whether clinical remission was accompanied by corresponding serological changes. Furthermore, serial laboratory safety monitoring, including complete blood counts and liver and renal function tests, was not performed, limiting the assessment of treatment safety beyond clinically apparent adverse events. Multicenter collaborative studies collecting real-world data from additional cases are therefore warranted to further elucidate the efficacy, safety and optimal dosing of nicotinamide for IBP.

In conclusion, IBP should be included in the differential diagnosis for infants and young children presenting with pruritic, tense vesicles and bullae, especially those with predominant acral involvement. Accurate diagnosis relies on the comprehensive combination of clinical manifestations, histopathological examination, DIF staining, and BP180-specific serological testing. In our two patients, complete clinical remission was observed following the initiation of nicotinamide monotherapy, with no recurrence during long-term follow-up. These cases provide preliminary clinical experience with nicotinamide monotherapy in IBP, and further studies are needed to determine its efficacy, safety, optimal dosage, and potential role in the management of IBP.

## Data Availability

The original contributions presented in this case report are included in the article. Further inquiries can be directed to the corresponding author.
